# Biomarker-based profiling of fatigue in childhood cancer survivors: evidence for distinct inflammatory and glial-associated profiles

**DOI:** 10.1016/j.bbih.2025.101089

**Published:** 2025-08-11

**Authors:** Deveny Vanrusselt, Sabine Verschueren, Lize Van Meerbeeck, Jurgen Lemiere, Stephanie Humblet-Baron, Charlotte Sleurs, Anne Uyttebroeck

**Affiliations:** a Department of Oncology, KU Leuven, Leuven, Belgium; b Pediatric Hematology and Oncology, University Hospitals Leuven, Leuven, Belgium; c Department of Rehabilitation Sciences, KU Leuven, Leuven, Belgium; d Department of Adaptive Immunology, KU Leuven, Leuven, Belgium; e Department of Social and Behavioral Sciences, Tilburg University, Tilburg, the Netherlands

**Keywords:** Astrocytic processes, Biomarkers, Childhood cancer survivors, Fatigue, Fatigue profiles, Systemic inflammation

## Abstract

**Background:**

Fatigue is a prevalent and burdensome late effect in childhood cancer survivors (CCS), yet its biological underpinnings remain poorly understood. This study examined associations between fatigue and blood-based biomarkers in CCS compared to healthy controls (HCs) and explored whether biologically distinct CCS profiles with respect to fatigue could be identified.

**Procedure:**

Eighty CCS (aged 14–28) and 35 age- and sex-matched HCs provided blood samples and completed the Pediatric Quality of Life Inventory Multidimensional Fatigue Scale (PedsQL-MFS). Plasma concentrations of 12 biomarkers (e.g., IL-2, TNF-α, BDNF, Total Tau, NfL, MCP-1, GFAP) were quantified using Meso Scale Discovery immunoassays. Analyses included group comparisons, Spearman correlations, and unsupervised clustering (hierarchical and k-means).

**Results:**

CCS reported significantly higher fatigue than HCs and showed significantly elevated levels of GFAP (d = 0.43), MCP-1 (d = 0.74), and Total Tau (d = 0.54). No individual biomarkers differentiated fatigued from non-fatigued CCS. Clustering revealed two biomarker-based CCS subgroups: one with high levels of inflammatory and neurodegenerative markers, and one with lower levels, yet fatigue severity was comparable. Within-cluster analyses showed distinct patterns: in the low-biomarker group, fatigue was associated with GFAP (ρ = −0.26 and ρ = −0.27, p < 0.05), whereas in the high-biomarker group, fatigue was more consistently linked to IL-8, IL-1α, and TNF-α (ρ = −0.38 to −0.49, p < 0.05).

**Conclusion:**

Findings suggest that fatigue in CCS may be associated with distinct biological pathways, including astrocyte-linked processes in one subgroup and systemic inflammation in another. This suggests the need for more personalized, biomarker-informed strategies to understand and manage fatigue in pediatric cancer survivorship.

**Table:** ABBREVIATIONS

ANOVA	Analysis of Variance
BDNF	Brain-Derived Neurotrophic Factor
βNGF	Beta Nerve Growth Factor
CCS	Childhood Cancer Survivors
CI	Confidence Interval
CNS	Central Nervous System
CV	Coefficient of Variation
EDTA	Ethylenediaminetetraacetic Acid
GFAP	Glial Fibrillary Acidic Protein
HCs	Healthy Controls
IL-1α	Interleukin-1 Alpha
IL-2	Interleukin-2
IL-8	Interleukin-8
IL-12p70	Interleukin-12 p70
IQR	Interquartile Range
Max	Maximum
MCP-1	Monocyte Chemoattractant Protein-1
Min	Minimum
MSD	Meso Scale Discovery
NfL	Neurofilament Light Chain
PedsQL-MFS	Pediatric Quality of Life Inventory Multidimensional Fatigue Scale
RT	Radiotherapy
SD	Standard Deviation
TNF- α	Tumor Necrosis Factor-Alpha
Total Tau	Total Tau Protein
VEGF(-A)	Vascular Endothelial Growth Factor (-A)

## 1 Introduction

Advances in pediatric oncology have led to long-term survival for approximately 80 % of children and adolescents diagnosed with cancer (Gatta et al., 2009). However, many survivors experience late effects, with fatigue being among the most common and burdensome. Fatigue can persist for years and significantly impact daily functioning, academic performance, and overall quality of life (Penson et al., 2022). Despite its impact, the etiology of fatigue in childhood cancer survivors (CCS) remains poorly understood, complicating clinical management and targeted intervention development (Levesque et al., 2022). To move toward more personalized fatigue management, there is growing interest in identifying biological markers that reflect underlying mechanisms and may guide tailored interventions.

Promising candidates include markers of inflammation and neurobiological function. Cancer treatments may trigger persistent inflammation, resembling inflammaging observed in older populations, marked by elevated levels of cytokines including tumor necrosis factor-alpha (TNF-α), interleukin (IL)-1α, IL-2, IL-8, and IL-12p70 (Xia et al., 2016; Rossi et al., 2021). These have been associated with fatigue in adult cancer populations and chronic conditions such as myalgic encephalomyelitis/chronic fatigue syndrome and COVID-19 (Tilikete et al., 2024; Kurzrock, 2001; Montoya et al., 2017). Other relevant markers are monocyte chemoattractant protein-1 (MCP-1), involved in immune cell recruitment (Deshmane et al., 2009), and vascular endothelial growth factor (VEGF), which affects vascular permeability and neuroinflammatory respones (Takahashi and Shibuya, 2005), and have likewise been linked to fatigue in adults with cancer, fibromyalgia, and chronic fatigue syndrome (Himbert et al., 2019; Groven et al., 2020). Markers of neuronal injury and glial reactivity, including neurofilament light chain (NfL), glial fibrillary acidic protein (GFAP), brain-derived neurotrophic factor (BDNF), and Total Tau have been linked to fatigue and cognitive dysfunction in neurological and oncological populations (Azcue et al., 2024; Desai et al., 2024; Henneghan et al., 2020). These may reflect neurotoxic effects of cancer therapies, especially central nervous system (CNS)-directed treatments (Gust et al., 2023).

Heterogeneity of the survivor population may complicate the identification of robust biomarker–fatigue associations, as diagnosis type, treatment exposure, and time since diagnosis influence recovery and risk. Emerging evidence suggests that hematological malignancies and prolonged chemotherapy can induce sustained immune activation, while CNS-directed therapies could trigger glial reactivity, each contributing to fatigue via distinct biological pathways (Myers, 2008; Bower and Lamkin, 2013; Dietrich et al., 2015). To address these gaps, the present study aimed to (1) compare biomarker concentrations between CCS and healthy controls (HCs); (2) investigate associations between biomarkers, fatigue (in CCS and HCs) and cancer-related characteristics (in CCS), (3) identify biomarker-based CCS subgroups using clustering analysis and compare fatigue levels; and (4) explore fatigue–biomarker associations within clusters. By integrating biological and clinical data, this research seeks to improve the understanding of fatigue in CCS and pave the way for targeted interventions.

## 2 Methods

### 2.1 Study procedure

This cross-sectional study was conducted at University Hospitals Leuven (Belgium) and approved by the KULeuven/UZLeuven Medical Ethics Committee (B3222021000452), in accordance with the Declaration of Helsinki. Between 2021 and 2023, 80 Dutch-speaking CCS were recruited from the Pediatric Hemato-Oncology Department. Eligibility included any cancer diagnosis at ≤18 years, current age between 14 and 28, completion of chemotherapy and/or radiotherapy 6 months to 10 years prior. Survivors treated with surgery only, diagnosed depression, or genetic syndromes were excluded. Interested participants were invited by mail and phone, and scheduled for a hospital visit to complete the Pediatric Quality of Life Multidimensional Fatigue Scale (PedsQL-MFS) and provide a blood sample. A group of 35 HCs, matched to the CCS group at group level for age and sex, was recruited through flyers distributed in high schools, colleges, and universities. Inclusion criteria for HCs included being Dutch-speaking, aged 14–28 years, and without a history of cancer or chronic illness.

### 2.2 Measurements

#### 2.1.1 Biomarkers

Blood was drawn into EDTA tubes and centrifuged. The supernatant was isolated, aliquoted into 1 ml aliquots, snap frozen and stored at −80 °C. Plasma biomarkers were quantified in duplicate using validated Meso Scale Discovery (MSD; Rockville, MD) multiplex immunoassays. S-plex assays were used for GFAP, Total Tau and Nfl (K15639S-1); U-plex assays for MCP-1, Beta Nerve Growth Factor (βNGF), TNF-α, IL-1α, BDNF, IL-2, IL-8, IL-12p70 and VEGF (K151ACM-1). MSD assays were performed as per the manufacturer's instructions. A standard curve was generated by fitting the signals from the standards using a 4-parameter logistic model. Sample concentrations were calculated by interpolating the electrochemiluminescence signals on the standard curve and adjusting for the dilution factor. Measurements with >20 % coefficient of variation (CV) between duplicates were excluded.

#### 2.1.2 Fatigue

Fatigue was assessed using the 18-item PedsQL-MFS, with items rated on a 5-point scale (0 = never to 4 = almost always). Scores were reversed and converted to 0–100, with lower scores indicating higher fatigue. Total and subscale scores (general, sleep/rest, cognitive) were calculated (study-specific Cronbach's α = 0.89, test–retest reliability coefficient = 0.90) (Varni et al., 2004).

### 2.3 Statistical analysis

Biomarker distributions were assessed using the Shapiro–Wilk test. Outliers were removed using Tukey's fences. Normally distributed variables were reported as means (SD) and compared using t-tests; non-normal variables were summarized as medians (IQR) and tested using Mann–Whitney U. Effect sizes (Cohen's d) were calculated for significant biomarkers. Bonferroni corrections were applied to fatigue comparisons, resulting in an adjusted alpha level of 0.0125 (0.05/4 tests) but not to biomarker comparisons, given the exploratory nature of these analyses and the risk of overlooking meaningful biological signals. Spearman correlations were used to examine associations between biomarkers and fatigue (general, sleep/rest, cognitive, total), and between biomarkers and cancer-related variables (e.g., age at diagnosis, time since diagnosis, treatment duration and time between end of treatment and inclusion). CCS were also standardized and categorized into fatigued (z-score < −1) and non-fatigued groups per fatigue domain to explore biomarker differences by fatigue severity. To identify biologically distinct CCS subgroups and compare their fatigue levels, unsupervised clustering was applied to the standardized (z-scored) biomarker data (Sinaga and Yang, 2020). All biomarkers were included in the clustering analysis. Missing values were imputed with medians (4 % (n = 38) applied only to the biomarker data, not to the fatigue measures). The optimal number of clusters was determined using the Silhouette method. Hierarchical clustering (Ward's linkage method) was used to visualize participant grouping via dendrograms, followed by k-means clustering with 25 random starts to ensure robustness. Fatigue levels across clusters and HCs were compared using ANOVA and Tukey's post-hoc tests. Biomarker and fatigue profiles across CCS clusters and HCs were compared using ANOVA with Tukey's post-hoc tests. Cluster-specific Spearman correlations explored distinct fatigue–biomarker associations.

## 3 Results

### 3.1 Biomarker differences between childhood cancer survivors and healthy controls

All fatigue scales, Total Tau, GFAP, MCP-1, and βNGF were normally distributed, BDNF, NfL, IL-2, TNF-α, IL-1α, IL-8, IL-12p70, and VEGF were not. The sample included 80 CCS (52.50 % (n = 42) male), most treated with chemotherapy only (62.50 % (n = 50)), 10.00 % (n = 8) with RT only and 27.50 % (n = 22) with a combination. Diagnoses included hematological malignancies (48.75 % (n = 39)), solid tumors (27.50 % (n = 22)) and brain tumors (23.75 % (n = 19)). Table 1 shows CCS characteristics. Additionally, 35 HCs (age- and sex matched at group level) were included (51.50 % male (n = 18), median age: 18.82 years).

**Table 1:** Childhood cancer survivors characteristics.

Variable	Median	IQR	Min	Max
**Age (yrs)**	18.76	5.49	14.26	28.23
**Age at primary diagnosis** (yrs)	13.83	5.31	0.00	18.47
**Time since primary diagnosis** (yrs)	5.08	5.22	1.13	21.59
**Overall treatment time** (yrs)	0.72	1.53	0.10	4.47
**Time between end of treatment and inclusion** (yrs)	3.65	4.85	0.54	10.00

Abbreviations: IQR, interquartile range; Min, minimum; Max, maximum; yrs, years.

**Fig. 1:**
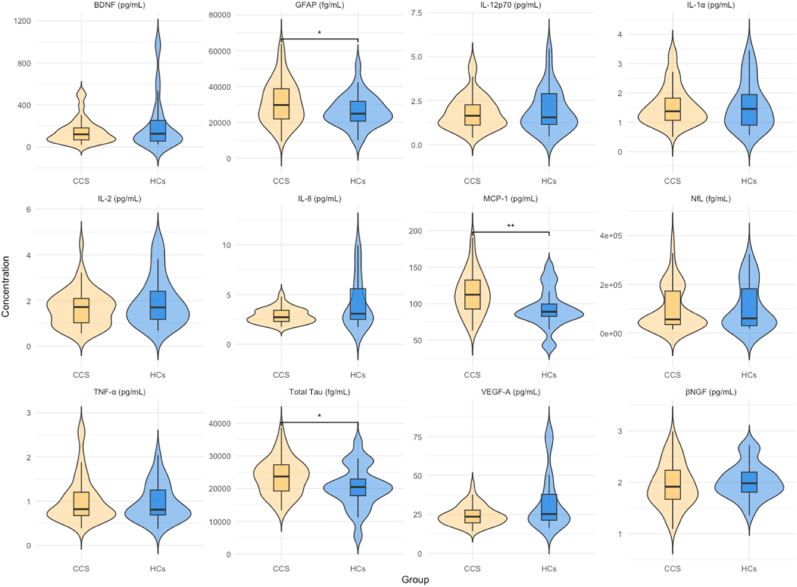
Violin plots of biomarkers concentrations per group (CCS and HCs). *Note.* Violin plots showing the distribution of biomarker concentrations for childhood cancer survivors (CCS) and healthy controls (HCs). Significant differences are indicated. ∗∗∗p < 0.001, ∗∗p < 0.01, ∗p < 0.05.

Fig. 1 visualizes biomarkers distributions between CCS and HCs; Table 2 presents descriptive statistics. CCS reported significantly more general, total, and cognitive fatigue than HCs (all p < 0.001). Biomarker comparisons showed significantly higher concentrations of GFAP (t(115) = 2.25 (p = 0.027)), MCP-1 (t(115) = 3.33 (p = 0.001)), and Total Tau (t(115) = 2.04 (p = 0.041)) in CCS compared to HCs. No other biomarkers differed significantly.

**Table 2:** Fatigue scores and biomarker concentrations in childhood cancer survivors and healthy controls.

Variable	Mean (SD)		Independent Sample T-Test
	CCS	HCs	
**General Fatigue**	61.11 (15.77)	74.70 (16.31)	t(115) = -3.81 (p < 0.001)∗
**Sleep/Rest Fatigue**	57.27 (17.30)	67.56 (15.02)	t(115) = -2.99 (p = 0.016)
**Cognitive Fatigue**	55.88 (21.37)	70.83 (17.57)	t(115) = -3.64 (p < 0.001)∗
**Total Fatigue**	58.08 (14.68)	71.03 (13.19)	t(115) = -4.32 (p < 0.001)∗
**Total Tau** (fg/mL)	23607.67 (5589.36)	20425.45 (6584.53)	t(115) = 2.04 (p = 0.041)∗
**GFAP** (fg/mL)	31213.69 (12080.06)	26364.85 (8943.47)	t(115) = 2.25 (p = 0.027)∗
**MCP-1** (pg/mL)	113.86 (29.31)	92.64 (26.43)	t(115) = 3.33 (p = 0.001)∗∗
**βNGF** (pg/mL)	1.95 (0.43)	2.03 (0.34)	t(115) = -0.92 (p = 0.36)

Variable	Median (IQR)		Mann–Whitney *U* test
	CCS	HCs	
**NfL** (fg/mL)	56238.27 (137597.36)	60322.31 (151516.79)	U = 881, p = 0.90
**TNF-α** (pg/mL)	0.82 (0.53)	0.81 (0.56)	U = 820, p = 0.73
**IL-1α** (pg/mL)	1.38 (0.76)	1.46 (1.05)	U = 1073, p = 0.61
**BDNF** (pg/mL)	120.23 (112.87)	125.93 (195.94)	U = 888, p = 0.98
**IL-2** (pg/mL)	1.72 (1.06)	1.70 (1.23)	U = 602, p = 0.38
**IL-8** (pg/mL)	2.72 (1.13)	3.07 (3.09)	U = 550, p = 0.17
**IL-12p70** (pg/mL)	1.64 (1.15)	1.55 (1.76)	U = 838, p = 0.09
**VEGF-A** (pg/mL)	23.56 (8.10)	25.21 (16.57)	U = 843, p = 0.08

Abbreviations: CCS, childhood cancer survivors; HCs, healthy controls; SD, standard deviation; IQR, interquartile range; GFAP, glial fibrillary acidic protein; MCP-1, monocyte chemoattractant protein-1; βNGF, beta nerve growth factor; NfL, neurofilament light chain; TNF-α, tumor necrosis factor-alpha; IL-1α, interleukin-1 alpha; BDNF, brain-derived neurotrophic factor; IL-2, interleukin-2; IL-8, interleukin-8; IL-12p70, interleukin-12 p70; VEGF-A, vascular endothelial growth factor-A; fg/mL, femtograms per milliliter; pg/mL, picograms per milliliter.

∗p < 0.0125 (Bonferroni correction) for fatigue scales.

∗p < 0.05, ∗∗p < 0.01 for biomarkers.

Effect sizes calculations revealed a moderate effect for Total Tau (d = 0.54, 95 % CI [0.44, 0.65]), a small to moderate effect for GFAP (d = 0.43, 95 % CI [0.33, 0.53]), and a moderate-to-large effect MCP-1 (d = 0.74, 95 % CI [0.64, 0.85]). A forest plot visualizing the effect sizes is shown in Fig. 1 in the Supplementary File.

In CCS, IL-12p70 showed a weak significant positive correlation with overall treatment time (ρ = 0.30, *p* = 0.020), while βNGF (ρ = −0.27, p = 0.023) and Total Tau (ρ = −0.32, p = 0.010) exhibited weak significant negative correlations with time since primary diagnosis. Additionally, Total Tau was negatively and significantly correlated with the time between end of treatment and inclusion (ρ = −0.25, p = 0.043). Only one significant biomarker-fatigue correlation was found between BDNF levels and cognitive fatigue in HCs (ρ = 0.580, p = 0.025). In CCS no significant correlations were found between biomarker levels and fatigue domains (full correlation tables (Table 1, Table 2, Table 3) in Supplementary File). Furthermore, no significant differences in any of the biomarkers were observed when comparing fatigued and non-fatigued CCS across the four fatigue domains (violin plots per biomarker and number per group for fatigued and non-fatigued CCS shown in Fig. 2 in Supplementary File).

**Table 3:** Fatigue and biomarker comparisons between childhood cancer survivor clusters and healthy controls using ANOVA and Kruskal–Wallis Test.

Normally Distributed Variables – ANOVA								
Variable	Statistic	p-value	Cluster 1 (mean ± SD) (n = 56)	Cluster 2 (mean ± SD) (n = 24)	HCs (mean ± SD) (n = 35)	Cluster 2 – Cluster 1	HC – Cluster 1	HC – Cluster 2
**General Fatigue**	7.44	<0.001∗∗∗	61.03 ± 15.97	61.28 ± 15.66	74.70 ± 16.31	0.998	0.001∗∗	0.009∗∗
**Sleep/Rest Fatigue**	3.88	0.024∗	57.48 ± 17.85	56.77 ± 16.34	67.56 ± 15.02	0.984	0.031∗	0.059
**Cognitive Fatigue**	5.47	0.006∗∗	56.10 ± 20.76	55.38 ± 23.13	70.83 ± 17.57	0.989	0.007∗∗	0.022∗
**Total Fatigue**	8.36	<0.001∗∗∗	58.20 ± 14.65	57.81 ± 15.08	71.03 ± 13.19	0.993	<0.001∗∗∗	0.004∗∗
**Total Tau** (fg/mL)	3.41	0.038∗	22951.53 ± 5709.68	25044.92 ± 5156.07	20425.40 ± 6584.53	0.363	0.220	0.029∗
**GFAP** (fg/mL)	2.00	0.140	30936.12 ± 11513.21	31829.17 ± 13504.86	26364.85 ± 8943.47	0.947	0.191	0.195
**βNGF** (pg/mL)	4.04	0.021∗	1.87 ± 0.41	2.15 ± 0.42	2.03 ± 0.34	0.022∗	0.196	0.513
**MCP-1** (pg/mL)	7.99	<0.001∗∗∗	108.22 ± 24.60	124.89 ± 34.86	92.64 ± 26.43	0.057	0.070	<0.001∗∗∗

Non-Normally Distributed Variables – Kruskal Wallis								
Variable	Statistic	p-value	Cluster 1 (Median (IQR))	Cluster 2 (Median (IQR))	HCs (Median (IQR))	Cluster 2 – Cluster 1	HC – Cluster 1	HC – Cluster 2
**BDNF** (pg/mL)	4.57	0.102	93.15 (104.05)	162.32 (156.89)	125.93 (195.94)	0.098	0.975	0.606
**NfL** (fg/mL)	0.59	0.744	48589.41 (94507.49)	62555.03 (167506.10)	60322.31(151516.79)	0.999	0.989	0.958
**IL-2** (pg/mL)	21.28	<0.001∗∗∗	1.27 (0.91)	2.38 (0.68)	1.7 (1.23)	<0.001∗∗∗	0.036∗	0.051
**TNF-α** (pg/mL)	9.26	0.010∗∗	0.74 (0.40)	1.20 (0.77)	0.81 (0.56)	0.008∗∗	0.986	0.058
**IL-1α** (pg/mL)	34.37	<0.001∗∗∗	1.20 (0.39)	2.21 (0.83)	1.46 (1.04)	<0.001∗∗∗	0.293	<0.001∗∗∗
**IL-8** (pg/mL)	5.44	0.066	2.64 (0.85)	3.32 (1.00)	3.07 (3.09)	0.182	0.142	0.987
**IL-12p70** (pg/mL)	22.39	<0.001∗∗∗	1.27 (0.75)	2.66 (0.90)	1.55 (1.75)	<0.001∗∗∗	0.215	0.011∗
**VEGF-A** (pg/mL)	5.44	0.066	22.96 (8.43)	24.72 (8.96)	25.21 (16.57)	0.340	0.091	0.998

Abbreviations: ANOVA, analysis of variance; CCS, childhood cancer survivors; HCs, healthy controls; SD, standard deviation; IQR, interquartile range; GFAP, glial fibrillary acidic protein; MCP-1, monocyte chemoattractant protein-1; βNGF, beta nerve growth factor; NfL, neurofilament light chain; TNF-α, tumor necrosis factor-alpha; IL-1α, interleukin-1 alpha; BDNF, brain-derived neurotrophic factor; IL-2, interleukin-2; IL-8, interleukin-8; IL-12p70, interleukin-12 p70; VEGF-A, vascular endothelial growth factor A; fg/mL, femtograms per milliliter; pg/mL, picograms per milliliter.

∗p < 0.05.

∗∗p < 0.01.

∗∗∗p < 0.001.

IQR values analyzed with Kruskal–Wallis test and Bonferroni-adjusted Dunn post hoc comparisons.

Means and SD analyzed with one-way ANOVA and Tukey post hoc tests.

**Fig. 2:**
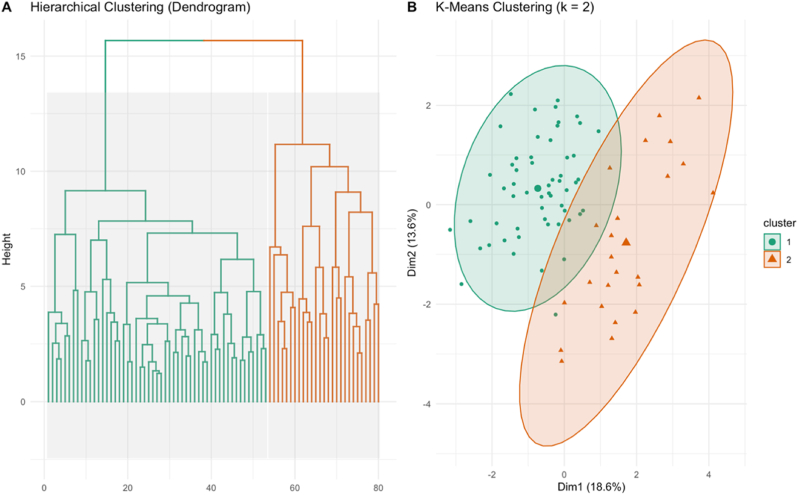
Biomarker-Based Subgrouping of Childhood Cancer Survivors Using Clustering Analyses. **(*A)****Hierarchical clustering dendrogram showing two distinct biomarker-based clusters among CCS.****(B)****K-means clustering (k = 2) visualized in reduced dimensional space, confirming separation between the identified subgroups. Cluster 1 (n = 56), Cluster 2 (n = 24).*

### 3.2 Identifying biomarker subgroups

To identify subgroups within CCS based on biomarker profiles, unsupervised clustering was applied. Hierarchical clustering (Ward's linkage method), generating a dendrogram, revealed two main clusters (Fig. 2A). Each branch of the dendrogram represents one CCS, with similar biomarker profiles grouped more closely together, indicating potential biological subgroupings. To validate and refine this grouping, k-means clustering (k = 2) was applied, which assigns each participant to one of two clusters based on proximity in multidimensional biomarker space. Based on this clustering, Cluster 1 consisted of 56 CCS and Cluster 2 included 24 CCS. The resulting cluster plot (Fig. 2B) shows the two clusters with minimal overlap, further supporting the presence of two biologically distinct subgroups within the CCS population. The Silhouette method indicated that a two-cluster solution provided the best balance of cohesion and separation (Fig. 3 in Supplementary File). Although both clustering methods produced similar subgroup structures, k-means clustering was used in subsequent analyses due to its direct assignment of individuals to groups and ease of interpretability. This allowed for more straightforward comparisons of fatigue and biomarker patterns between clusters.

**Fig. 3:**
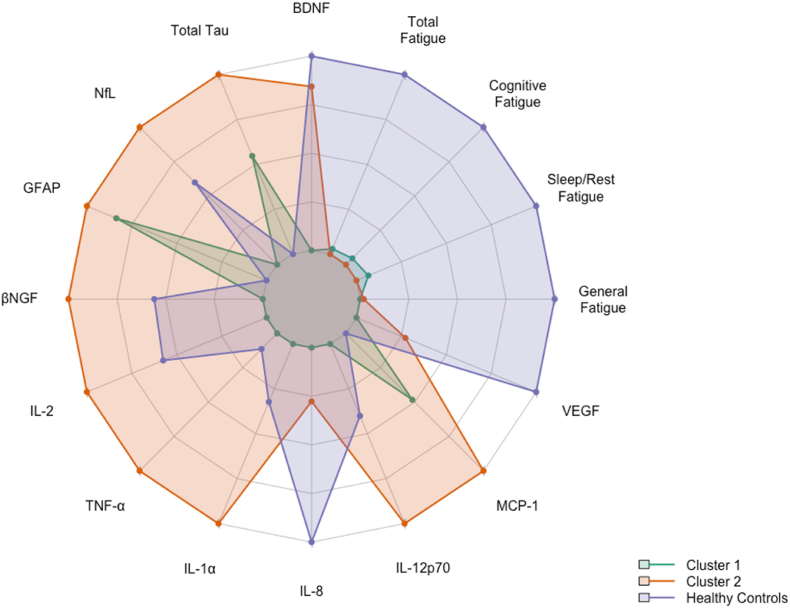
Comparative Biomarker and Fatigue Profiles Across CCS Clusters and Healthy Controls. Radar plot showing standardized mean values of fatigue domains (PedsQL-MFS) and blood biomarkers for Cluster 1 and Cluster 2 of childhood cancer survivors (CCS), as well as healthy controls (HCs). While HCs consistently report lower fatigue and lower inflammatory marker levels, CCS clusters differ primarily in biomarker expression, with similar fatigue severity. Note that PedsQL-MFS fatigue scores are inversely scaled, with lower scores indicating higher levels of fatigue.

The radar plot in Fig. 3 displays standardized mean values of 12 blood-based biomarkers (BDNF, Total Tau, NfL, GFAP, βNGF, IL-2, TNF-α, IL-1α, IL-8, IL-12p70, MCP-1, VEGF) and four fatigue scores (General, Sleep/Rest, Cognitive, and Total fatigue) across three groups: Cluster 1 (green), Cluster 2 (orange), and healthy controls (HCs; purple). All values are scaled from 0 (inner ring) to 1 (outer ring), enabling direct visual comparison across variables.

Healthy controls showed the most favorable profile: low fatigue, high BDNF and VEGF, low GFAP and Total Tau and generally low concentrations of pro-inflammatory cytokines (except for mid-to high-range IL-2 and IL-8) Detailed concentrations and scores per cluster can be found in Table 3. Fatigue severity was similarly high across both CCS clusters, despite differing biomarker profiles. Cluster 1 (green) had a low-biomarker/low-inflammatory profile, with reduced levels of pro-inflammatory cytokines (IL-2, TNF-α, IL-1α, IL-12p70), neurodegeneration-related markers (Total Tau, NfL, MCP-1), and lower BDNF and VEGF compared to Cluster 2 and HCs. Cluster 2 (orange) showed a high-biomarker/high-inflammatory profile, with elevated concentrations across most biomarkers. Fig. 4 in supplementary file shows violin plots illustrating biomarker concentrations and fatigue scores across Cluster 1, Cluster 2, and HCs.

Within-cluster correlations (Table 4in supplementary file) revealed that fatigue was largely uncorrelated with biomarkers in Cluster 1 (low-biomarker/low-inflammatory profile), except for significant negative associations between GFAP and both Sleep/Rest Fatigue (ρ = −0.27, p = 0.04) and Total Fatigue (ρ = −0.26, p = 0.03). A negative trend was also observed between Cognitive Fatigue and TNF-α (ρ = −0.28, p = 0.05). In Cluster 2 (high-biomarker/high-inflammatory profile), fatigue correlated more strongly with inflammation. Cognitive Fatigue correlated with IL-8 (ρ = −0.48, p = 0.02) and TNF-α (ρ = −0.49, p = 0.04); Total Fatigue also correlated with TNF-α (ρ = −0.49, p = 0.04), while General and Sleep/Rest Fatigue were associated with IL-1α (ρ = −0.42, p = 0.03 and ρ = –0.38, p = 0.04). When comparing the two CCS clusters to HCs, results showed that Cluster 1 was more similar to HCs in biomarker expression, particularly for Total Tau, MCP-1, and βNGF, where post-hoc comparisons showed smaller or non-significant differences between Cluster 1 and HCs. In contrast, Cluster 2 differed significantly from HCs across several biomarkers (higher levels of IL-1α, IL-12p70 and MCP-1), suggesting a more distinct inflammatory profile. However, fatigue levels were comparably high in both CCS clusters. Clinical characteristics were largely comparable between the two biomarker-based CCS clusters. Median age at primary diagnosis was similar between clusters (Cluster 1: 13.72 years, IQR = 3.94; Cluster 2: 13.71 years, IQR = 7.27; *p* = 0.856). Time since primary diagnosis (Cluster 1: 5.02 years, IQR = 5.62; Cluster 2: 6.18 years, IQR = 5.02; *p* = 0.502) and time between end of treatment and inclusion (Cluster 1: 4.14 years, IQR = 5.34; Cluster 2: 3.24 years, IQR = 3.49; *p* = 0.558) also did not significantly differ between clusters. However, Cluster 2 showed a significantly longer overall treatment duration (median = 1.25 years, IQR = 1.26) compared to Cluster 1 (median = 0.63 years, IQR = 0.86), *p* = 0.007. Treatment modality distributions were also examined across clusters. In Cluster 1, the majority of survivors (58.90 %) received chemotherapy only, followed by 30.40 % who received combined therapy (chemotherapy and radiotherapy), and 10.70 % who received radiotherapy only. In Cluster 2, 66.70 % received chemotherapy only, 25.00 % received combined therapy, and 6.30 % were treated with radiotherapy only. Diagnosis group distributions were also examined across clusters. In Cluster 1, 41.10 % of survivors were diagnosed with a hematological malignancy, 26.80 % with a brain tumor, and 32.10 % with a solid tumor. In Cluster 2, 66.60 % had a hematological malignancy, 16.70 % had a brain tumor, and 16.70 % had a solid tumor. No significant differences were found in the distribution of therapy types (*p* = 0.93) or diagnosis groups (*p* = 0.13) between clusters (Fisher's exact tests).

## 4 Discussion

This exploratory study investigated the relationship between fatigue and a panel of biomarkers in CCS compared to HCs, offering new insights into the biological underpinnings of fatigue in CCS. Consistent with prior research, fatigue was significantly more severe in CCS (Penson et al., 2023; Vanrusselt et al., 2025). By combining biomarker analysis with unsupervised clustering, this study identified group-level biomarker differences and distinct biological subgroups within CCS. The findings suggest that fatigue may not reflect a single biological pathway but instead arises from heterogeneous neuroimmune profiles within the survivor population.

CCS showed elevated GFAP, MCP-1, and Total Tau compared to HCs, suggesting persistent neuroimmune alterations, possibly reflecting the long-term biological impact of cancer and its treatment. These finding align with prior (preclinical) studies linking GFAP, a marker of astrocyte activation and neuroinflammation, to neuroinflammatory processes following chemotherapy or radiotherapy (McLeary et al., 2019; Fernström et al., 2024). MCP-1 supports evidence of chronic immune activation (Singh et al., 2021), and has been associated with fatigue and cognitive symptoms in adult cancer populations, making it a candidate biomarker for treatment-related immune dysregulation (Janelsins et al., 2022; Lyon et al., 2016). 10.13039/501100007185Total Tau, a well-established marker of neurodegeneration, reflects neuronal injury or delayed neural recovery and is consistent with studies reporting Tau elevations after CNS-directed therapies (Cheung et al., 2018; Muszyńska-Rosłan et al., 2006). IL-12p70 showed a significant weak positive correlation with overall treatment time, possibly indicating low-grade immune activation persisting in CCS with longer treatment histories (Ullrich et al., 2020). While the negative correlation between 10.13039/501100007185Total Tau and time since primary diagnosis as well as with time since end of treatment, may point to slow recovery or reorganization of neuronal processes following initial cancer- and treatment-related injury, the interpretation of the negative correlation between β10.13039/100017798NGF and time since primary diagnosis is less straightforward. As a nerve growth factor involved in neuronal survival and protection**,** axonal growth, synaptic plasticity, and immune modulation, β10.13039/100017798NGF is generally considered protective (Skaper, 2017). Therefore, lower β10.13039/100017798NGF levels with increasing time since diagnosis might reflect a decline in neurotrophic support during the survivorship phase, possibly due to long-term neurobiological adaptations or reduced compensatory mechanisms post-treatment. The functional implications of this decrease remain unclear, but they may point to subtle changes in neural plasticity or inflammatory regulation in long-term survivors. Only one significant biomarker-fatigue correlation emerged: in HCs, lower BDNF was linked to greater cognitive fatigue. BDNF is essential for neurogenesis, learning, and memory, and reduced peripheral BDNF has been implicated in fatigue and mood disturbances in clinical populations (Li et al., 2022; Castrén and Monteggia, 2021; Hashimoto, 2010). In CCS, this association was absent, suggesting that fatigue in this population may reflect more complex or treatment-related biological mechanisms (Bower, 2014).

No single biomarker distinguished fatigued from non-fatigued CCS, suggesting that while certain biomarkers may differentiate CCS from healthy individuals, they do not clearly distinguish between CCS subgroups based on fatigue severity using a binary cut-off (z-score < −1). It is possible that individual fatigue symptoms are not reflected in single biomarker levels, but rather emerge from complex biological interactions that are better captured by biomarker combinations or profiles. Therefore, we applied a data-driven clustering approach and identified two biologically distinct CCS subgroups with comparable fatigue severity. Cluster 1 exhibited a low-biomarker/low-inflammatory profile, characterized by reduced levels of pro-inflammatory cytokines and neurodegeneration-related markers. It is noteworthy that survivors in Cluster 1 exhibited lower levels of several inflammatory markers compared to healthy controls, yet still reported higher levels of fatigue. This counterintuitive finding challenges the assumption that fatigue in cancer survivors is always driven by heightened inflammation. Instead, these lower levels of inflammatory markers may reflect a state of suppressed immune signaling, potentially resulting from immune exhaustion, immune senescence, or delayed immune reconstitution following cancer and treatment (Zhao et al., 2020). An alternative or complementary explanation may lie in localized neuroglial dysregulation. Besides the interleukines, βNGF was also lower in Cluster 1. Given βNGF's role as a neurotrophic factor involved in neuronal survival, synaptic plasticity, and modulation of immune responses, lower levels in Cluster 1 may indicate reduced neurotrophic support in survivors with low systemic inflammation. This could suggest that in the absence of heightened peripheral inflammation, mechanisms related to impaired neuronal repair or reduced neuroprotective signaling may play a more prominent role in fatigue pathophysiology (Petrella et al., 2022; Capossela et al., 2024). Within this Cluster 1, higher GFAP were associated with higher fatigue, suggesting that localized astrocytic involvement may be associated with fatigue in the absence of systemic inflammation. This aligns with recent findings. For instance, studies have shown that elevated GFAP levels are associated with neurological symptoms such as fatigue and cognitive impairment in patients recovering from COVID-19 (Costanza et al., 2024; Kanberg et al., 2021). Additionally, increased GFAP concentrations have been observed in individuals with major depressive disorder, correlating with disease severity (Codeluppi et al., 2023; Miguel-Hidalgo, 2022). These findings support the notion that astrocytic dysfunction contributes to fatigue via changes in metabolic support, neurotransmitter clearance, or neuroimmune interactions. In contrast, Cluster 2 showed a high-biomarker/high-inflammatory profile with elevated levels across most pro-inflammatory cytokines as well as markers of neurodegeneration. Fatigue in this group was more consistently linked to inflammatory markers. These findings are in line with a large body of literature in adult cancer and chronic illness populations linking fatigue to peripheral inflammation and cytokine dysregulation. For instance, Bower et al. (2011) showed that breast cancer survivors with persistent fatigue had elevated levels of inflammatory markers including TNF-α and IL-6, and Capuron et al. (2012) demonstrated associations between cytokine levels and fatigue severity in patients receiving interferon-α therapy (Capuron et al., 2011; Bower et al., 2011). Interestingly, despite elevated GFAP concentrations in Cluster 2, no significant association was observed between GFAP and fatigue within this subgroup. This suggests that the role of GFAP in fatigue may be context-dependent, potentially influenced by the broader inflammatory environment. In Cluster 1, characterized by low systemic inflammation, GFAP may reflect localized astroglial activation that more directly contributes to fatigue symptoms. In Cluster 2, GFAP elevations may be part of a broader neuroimmune activation pattern, where its specific contribution is overshadowed by more dominant systemic inflammatory signals. Alternatively, fatigue in Cluster 2 may be less influenced by astrocytic mechanisms and more by widespread cytokine dysregulation. These differences underscore the heterogeneity of fatigue-related biology, even among survivors with similar symptom severity.

These results support the presence of biologically meaningful subtypes among CCS, characterized by distinct neurobiological profiles. While fatigue severity was similar across subgroups, one cluster showed patterns suggestive of astrocytic/glial involvement, and another was marked by systemic inflammation. To further contextualize these biological profiles, we compared both clusters to healthy controls and examined cancer-related characteristics. Cluster 1 was more similar to HCs in the majority of biomarker expression, while Cluster 2 showed greater elevations, particularly in inflammatory markers, yet fatigue severity remained comparable between the two clusters. Clinically, clusters were largely similar, though Cluster 2 had longer treatment durations and more survivors treated with chemotherapy only and diagnosed with hematological malignancies, an expected pattern given that hematological cancers are often managed with prolonged chemotherapy protocols. Cluster 1 had more survivors treated with combined chemo-radiotherapy and more brain or solid tumors. These differences, although insignificant, might indicate that fatigue arises through different neurobiological pathways depending on diagnosis and treatment history. This interpretation aligns with previous research showing that hematological malignancies and prolonged chemotherapy can lead to chronic immune activation (Cluster 2), while CNS-directed therapies in brain or solid tumors may induce glial responses or neuroinflammation (Cluster 1) (Bower, 2014; Monje and Dietrich, 2012; Garg, 2013; Krull et al., 2013).

While our findings offer valuable insights, several limitations should be acknowledged. The cross-sectional design limits causal interpretation. Longitudinal and mediation studies are needed. Our biomarker panel focused on neuroinflammatory and neurodegenerative markers, excluding other possibly relevant mechanisms (e.g., HPA-axis, mitochondrial activity). Although powered to detect medium effect sizes (Cohen's *d* ≈ 0.5) with 80 % power at α = 0.05, unequal group sizes and the inability to stratify by diagnosis or treatment limit generalizability. Future studies should explore whether specific therapies, especially CNS-directed ones, are linked to distinct biomarker patterns. Although CCS and HCs were matched at group level, the smaller HC sample may have reduced statistical power for between-group comparisons, especially for biomarkers with high interindividual variability. Additionally, we did not apply corrections for multiple comparisons in our biomarker analyses. While this decision was intentional due to the exploratory nature of the study, it increases the risk of Type I error and the possibility of false-positive findings. These results should therefore be interpreted as hypothesis-generating and require replication in future studies. While several biomarkers were elevated in Cluster 2, few showed direct associations with clinical fatigue levels. It is possible that these biomarkers reflect underlying biological vulnerability or chronic immune activation that does not manifest directly as fatigue alone, but may contribute to a broader spectrum of late effects. Furthermore, given the multifactorial nature of fatigue, these biological signals likely interact with key psychosocial and behavioral contributors, including stress, anxiety, depression, and sleep quality, which are well-established predictors of fatigue but were not assessed in this study. These factors may confound or mediate the observed biomarker–fatigue associations, and their integration in future research is essential to fully understand the interplay between biological and psychological mechanisms underlying fatigue. While some observed differences were statistically significant, the clinical relevance of these biomarker patterns remains uncertain. Future studies should examine whether these biologically defined subgroups differ in terms of functional outcomes, prognosis, or response to intervention.

In conclusion, this study provides new insights into the biological correlates of fatigue in CCS by integrating biomarker profiling with exploratory clustering. While fatigue was more severe in CCS and certain biomarkers (GFAP, MCP-1, Total Tau) were elevated, no single marker explained fatigue severity. Instead, two biologically distinct subtypes emerged, one marked by low inflammation but glial involvement, and another by systemic inflammatory activity. These patterns suggest that fatigue in survivors may be linked to heterogeneous neurobiological mechanisms, including astrocyte-associated or glial-enriched profiles and inflammation-related pathways. Our findings highlight the potential of biomarker-based subgrouping for understanding fatigue in cancer and underscore the need for personalized, multimodal approaches to its assessment and management in survivorship care. These exploratory findings lay the groundwork for future biomarker-informed approaches to fatigue in CCS, but clinical translation will depend on replication, external validation, and demonstration of differential outcomes across subgroups.

## CRediT authorship contribution statement

**Deveny Vanrusselt:** Writing – original draft, Visualization, Methodology, Investigation, Formal analysis, Conceptualization. **Sabine Verschueren:** Writing – review & editing, Supervision, Resources, Funding acquisition, Conceptualization. **Lize Van Meerbeeck:** Writing – review & editing, Resources, Formal analysis. **Jurgen Lemiere:** Writing – review & editing, Supervision, Conceptualization. **Stephanie Humblet-Baron:** Writing – review & editing, Supervision, Resources, Conceptualization. **Charlotte Sleurs:** Writing – review & editing, Supervision, Resources, Methodology, Conceptualization. **Anne Uyttebroeck:** Writing – review & editing, Visualization, Resources, Funding acquisition, Conceptualization.

## Patient consent statement

All participants, or their legal guardians where applicable, provided written informed consent prior to participation in the study.

## Ethics approval

This study was performed in line with the principles of the Declaration of Helsinki. Approval was granted by the Medical Ethics Committee of KU Leuven/UZ Leuven (Belgium - B3222021000452).

## Funding

This work was supported by 10.13039/501100003130Fonds Wetenschappelijk Onderzoek (FWO) —Kom Op Tegen Kanker (Grant G0D9621N; Prof. Dr. Anne Uyttebroeck), Emmanuel Van Der Schueren – Kom Op Tegen Kanker (Grant ref. 13887; Drs. Deveny Vanrusselt) and Kinderkankerfonds Leuven.

## Declaration of competing interest

The authors declare that they have no known competing financial interests or personal relationships that could have appeared to influence the work reported in this paper.

## Data Availability

Data will be made available on request.

## References

[bib1] Azcue N., Tijero-Merino B., Acera M. (2024). Plasma neurofilament light chain: a potential biomarker for neurological dysfunction in Myalgic Encephalomyelitis/Chronic fatigue syndrome. Biomedicines.

[bib2] Bower J.E. (2014). Cancer-related fatigue--mechanisms, risk factors, and treatments. Nat. Rev. Clin. Oncol..

[bib3] Bower J.E., Lamkin D.M. (2013). Inflammation and cancer-related fatigue: mechanisms, contributing factors, and treatment implications. Brain Behav. Immun..

[bib4] Bower J.E., Ganz P.A., Irwin M.R., Kwan L., Breen E.C., Cole S.W. (2011). Inflammation and behavioral symptoms after breast cancer treatment: do fatigue, depression, and sleep disturbance share a common underlying mechanism?. J. Clin. Oncol..

[bib5] Capossela L., Gatto A., Ferretti S. (2024). Multifaceted roles of nerve growth factor: a comprehensive review with a special Insight into pediatric perspectives. Biology.

[bib6] Capuron L., Schroecksnadel S., Féart C. (2011). Chronic low-grade inflammation in elderly persons is associated with altered tryptophan and tyrosine metabolism: role in neuropsychiatric symptoms. Biol. Psychiatry.

[bib7] Castrén E., Monteggia L.M. (2021). Brain-Derived neurotrophic factor signaling in depression and antidepressant action. Biol. Psychiatry.

[bib8] Cheung Y.T., Khan R.B., Liu W. (2018). Association of cerebrospinal fluid biomarkers of central nervous system injury with neurocognitive and brain imaging outcomes in children receiving chemotherapy for acute lymphoblastic leukemia. JAMA Oncol..

[bib9] Codeluppi S.A., Xu M., Bansal Y. (2023). Prefrontal cortex astroglia modulate anhedonia-like behavior. Mol. Psychiatr..

[bib10] Costanza A., Amerio A., Aguglia A. (2024). Reactive Astrocytosis—A potential contributor to increased suicide in long COVID-19 patients?. Brain Sci..

[bib11] Desai P., Krueger K.R., de Leon C.M., Wilson R.S., Evans D.A., Rajan K.B. (2024). Depressive symptoms, glial fibrillary acid protein concentrations, and cognitive decline in a cohort Study. Journals of Gerontology - Series A Biological Sciences and Medical Sciences.

[bib12] Deshmane S.L., Kremlev S., Amini S., Sawaya B.E. (2009). Monocyte chemoattractant protein-1 (MCP-1): an overview. J. Interferon Cytokine Res..

[bib13] Dietrich J., Prust M., Kaiser J. (2015). Chemotherapy, cognitive impairment and hippocampal toxicity. Neuroscience.

[bib14] Fernström E., Jarfelt M., Blomstrand M. (2024). CSF biomarkers of neurotoxicity in childhood cancer survivors after cranial radiotherapy or surgery. Ann Clin Transl Neurol.

[bib15] Garg A.K. (2013). Fatigue, inflammation, and ω-3 and ω-6 fatty acid intake among breast cancer survivors. Breast Dis. Year Bk. Q..

[bib16] Gatta G., Zigon G., Capocaccia R. (2009). Survival of European children and young adults with cancer diagnosed 1995-2002. Eur. J. Cancer.

[bib17] Groven N., Fors E.A., Stunes A.K., Reitan S.K. (2020). MCP-1 is increased in patients with CFS and FM, whilst several other immune markers are significantly lower than healthy controls. Brain Behav Immun Health.

[bib18] Gust J., Rawlings-Rhea S.D., Wilson A.L. (2023). GFAP and NfL increase during neurotoxicity from high baseline levels in pediatric CD19-CAR T-cell patients. Blood Adv..

[bib19] Hashimoto K. (2010). Brain-derived neurotrophic factor as a biomarker for mood disorders: an historical overview and future directions. Psychiatr. Clin. Neurosci..

[bib20] Henneghan A., Haley A.P., Kesler S. (2020). Exploring relationships among peripheral amyloid beta, Tau, cytokines, cognitive function, and psychosomatic symptoms in breast cancer survivors. Biol. Res. Nurs..

[bib21] Himbert C., Ose J., Lin T. (2019). Inflammation- and angiogenesis-related biomarkers are correlated with cancer-related fatigue in colorectal cancer patients: results from the ColoCare Study. Eur. J. Cancer Care.

[bib22] Janelsins M.C., Lei L., Netherby-Winslow C. (2022). Relationships between cytokines and cognitive function from pre- to post-chemotherapy in patients with breast cancer. J. Neuroimmunol..

[bib23] Kanberg N., Simrén J., Edén A. (2021). Neurochemical signs of astrocytic and neuronal injury in acute COVID-19 normalizes during long-term follow-up. EBioMedicine.

[bib24] Krull K.R., Brinkman T.M., Li C. (2013). Neurocognitive outcomes decades after treatment for childhood acute lymphoblastic leukemia: a report from the St jude lifetime cohort study. J. Clin. Oncol..

[bib25] Kurzrock R. (2001).

[bib26] Levesque A., Caru M., Duval M., Laverdière C., Marjerrison S., Sultan S. (2022). Cancer-related fatigue in childhood cancer survivors: a systematic scoping review on contributors of fatigue and how they are targeted by non-pharmacological interventions. Crit. Rev. Oncol. Hematol..

[bib27] Li Y., Li F., Qin D. (2022). The role of brain derived neurotrophic factor in central nervous system. Front. Aging Neurosci..

[bib28] Lyon D.E., Cohen R., Chen H. (2016). Relationship of systemic cytokine concentrations to cognitive function over two years in women with early stage breast cancer. J. Neuroimmunol..

[bib29] McLeary F., Davis A., Rudrawar S., Perkins A., Anoopkumar-Dukie S. (2019). Mechanisms underlying select chemotherapeutic-agent-induced neuroinflammation and subsequent neurodegeneration. Eur. J. Pharmacol..

[bib30] Miguel-Hidalgo J.J. (2022). Astroglia in the vulnerability to and maintenance of stress-mediated neuropathology and depression. Front. Cell. Neurosci..

[bib31] Monje M., Dietrich J. (2012). Cognitive side effects of cancer therapy demonstrate a functional role for adult neurogenesis. Behav. Brain Res..

[bib32] Montoya J.G., Holmes T.H., Anderson J.N. (2017). Cytokine signature associated with disease severity in chronic fatigue syndrome patients. Proc Natl Acad Sci.

[bib33] Muszyńska-Rosłan K., Krawczuk-Rybak M., Protas P.T., Hołownia A. (2006). Level of tau protein in children treated for acute lymphoblastic leukemia. Pediatr. Neurol..

[bib34] Myers J.S. (2008). Proinflammatory cytokines and sickness behavior: implications for depression and cancer-related symptoms. Oncol. Nurs. Forum.

[bib35] Penson A., Walraven I., Bronkhorst E. (2022). The impact of cancer-related fatigue on HRQOL in survivors of childhood cancer: a DCCSS LATER Study. Cancers (Basel).

[bib36] Penson A., Walraven I., Bronkhorst E. (2023). Chronic fatigue in childhood cancer survivors is associated with lifestyle and psychosocial factors; a DCCSS LATER study. ESMO Open.

[bib37] Petrella C., Nenna R., Petrarca L. (2022). Serum NGF and BDNF in Long-COVID-19 adolescents: a pilot Study. Diagnostics.

[bib38] Rossi F., Di Paola A., Pota E. (2021). Biological aspects of inflamm-aging in childhood cancer survivors. Cancers (Basel).

[bib39] Sinaga K.P., Yang M.S. (2020). Unsupervised K-means clustering algorithm. IEEE Access.

[bib40] Singh S., Anshita D., Ravichandiran V. (2021). MCP-1: function, regulation, and involvement in disease. Int. Immunopharmacol..

[bib41] Skaper S.D. (2017). Nerve growth factor: a neuroimmune crosstalk mediator for all seasons. Immunology.

[bib42] Takahashi H., Shibuya M. (2005). The vascular endothelial growth factor (VEGF)/VEGF receptor system and its role under physiological and pathological conditions. Clin. Sci..

[bib43] Tilikete C., Zamali I., Meddeb Z. (2024). Exploring the landscape of symptom-specific inflammatory cytokines in post-COVID syndrome patients. BMC Infect. Dis..

[bib44] Ullrich K.A.M., Schulze L Lou, Paap E.M., Müller T.M., Neurath M.F., Zundler S. (2020). Immunology of IL-12: an update on functional activities and implications for disease. EXCLI J.

[bib45] Vanrusselt D., Sleurs C., Van Ermengem N. (2025). Sleep quality and physical fitness as modifiable contributors of fatigue in childhood cancer survivors. Journal of Cancer Survivorship.

[bib46] Varni J.W., Burwinkle T.M., Szer I.S. (2004). The PedsQL^TM^ multidimensional fatigue scale in pediatric rheumatology: reliability and validity. J. Rheumatol..

[bib47] Xia S., Zhang X., Zheng S. (2016). An update on Inflamm-Aging: Mechanisms, prevention, and treatment. J Immunol Res.

[bib48] Zhao Y., Shao Q., Peng G. (2020). Exhaustion and senescence: two crucial dysfunctional states of T cells in the tumor microenvironment. Cell. Mol. Immunol..

