# Supplementary material for: Biomarker-based profiling of fatigue in childhood cancer survivors: evidence for distinct inflammatory and glial-associated profiles
Source: Brain Behav Immun Health. 2025 Aug 11;48:101089. doi: 10.1016/j.bbih.2025.101089 (PMC12362137; doi:10.1016/j.bbih.2025.101089)
Supplement: Multimedia component 1 [file mmc1.docx]

**Supplementary File**


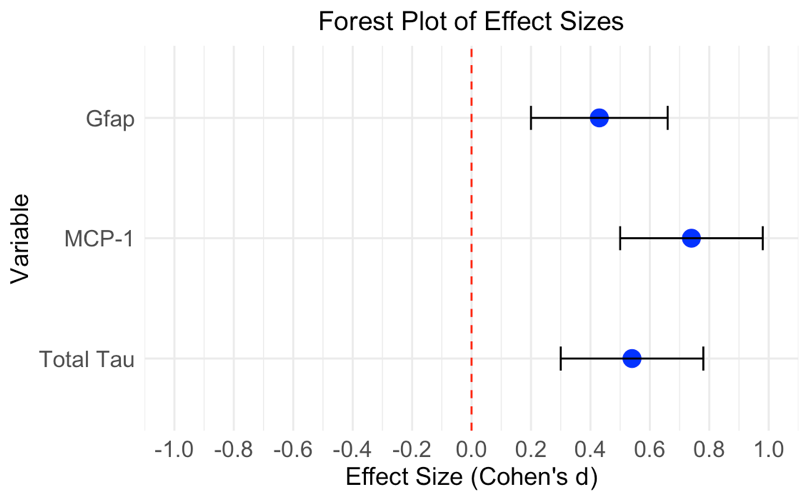
SUPPLEMENTARY FIGURE 1: Forest Plot of Effect Sizes for biomarkers significant differing between CCS and HCs.

Note. Forest plot depicting Cohen’s *d* effect sizes and 95% confidence intervals for GFAP, MCP-1, and Total Tau. All three biomarkers showed significantly higher levels in CCS compared to HCs. Effect sizes ranged from small to large, with MCP-1 showing the strongest group difference (d = 0.74), followed by Total Tau (d = 0.54) and GFAP (d = 0.43). The red dashed line represents zero effect (no group difference).

SUPPLEMENTARY TABLE 1: Spearman correlation matrix and p-values of cancer characteristics and blood-based biomarkers in childhood cancer survivors

|  | **Age** | **BMI** | **Age at primary diagnosis** | **Time since primary diagnosis** | **Overall treatment time** | **Time between end of treatment and inclusion** | **BDNF** | **Total Tau** | **NFL** | **GFAP** | **IL-2** | **TNF-α** | **IL-1α** | **IL-8** | **IL-12p70** | **MCP-1** | **VEGF-A** | **βNGF** |
| --- | --- | --- | --- | --- | --- | --- | --- | --- | --- | --- | --- | --- | --- | --- | --- | --- | --- | --- |
| **Age** | 1 | 0.27 (0.017)* | .55  (<0.001) *** | 0.24 (0.033)* | -0.01  (0.928) | 0.18  (0.105) | -0.21  (0.095) | -0.18  (0.153) | 0.08  (0.568) | -0.15 (0.212) | -0.03 (0.846) | -0.12 (0.379) | -0.08 (0.527) | -0.13 (0.348) | 0.10 (0.452) | 0.10 (0.441) | 0.002  (0.985) | -0.21 (0.078) |
| **BMI** |  | 1 | -0.006 (0.959) | 0.27  (0.018)* | -0.08 (0.481) | 0.16 (0.171) | -0.08 (0.528) | -0.06 (0.658) | -0.25 (0.062) | -0.45  (<0.001)*** | 0.16 (0.282) | 0.01 (0.930) | 0.12  (0.323) | -0.12 (0.381) | 0.35  (0.006)** | 0.10  (0.426) | 0.13 (0.299) | 0.13 (0.274) |
| **Age at primary diagnosis** |  |  | 1 | -0.57  (<0.001)*** | -0.06  (0.636) | -0.44  (<0.001)*** | -0.09  (0.455) | 0.16  (0.211) | -0.01  (0.980) | -0.19  (0.103) | -0.04  (0.807) | -0.03  (0.844) | -0.06  (0.653) | -0.21  (0.116) | 0.12  (0.374) | 0.080  (0.534) | -0.09  (0.471) | .017  (0.149) |
| **Time since primary diagnosis** |  |  |  | 1 | 0.12  (0.292) | 0.82  (<0.001)*** | 0.02  (0.906) | -0.32  (0.010)* | -0.04  (0.794) | 0.04  (0.757) | 0.05  (0.737) | -0.11  (0.444) | 0.17  (0.186) | 0.15  (0.252) | 0.04  (0.740) | 0.03  (0.797) | 0.17  (0.165) | -0.27  (0.023)* |
| **Overall treatment time** |  |  |  |  | 1 | -0.08  (0.474) | 0.07  (0.565) | 0.03  (0.844) | -0.06  (0.640) | 0.05  (0.653) | 0.04  (0.776) | 0.12  (0.413) | 0.10  (0.430) | 0.20  (0.132) | 0.30  (0.020)* | 0.05  (0.689) | -0.08  (0.534) | 0.16  (0.199) |
| **Time between end of treatment and inclusion** |  |  |  |  |  | 1 | 0.02  (0.874) | -0.25  (0.043)* | -0.01  (0.949) | 0.04  (0.723) | -0.04  (0.771) | -0.12  (0.376) | 0.04  (0.742) | -0.01  (0.960) | -0.21  (0.102) | -0.005  (0.971) | 0.11  (0.380) | -0.02  (0.897) |
| **BDNF** |  |  |  |  |  |  | 1 | 0.18  (0.182) | -0.005 (0.974) | -0.08  (0.519) | 0.07  (0.674) | 0.04  (0.808) | 0.20  (0.140) | -0.13  (0.376) | -0.02  (0.905) | 0.38  (0.003)** | 0.30  (0.021)* | 0.16  (0.237) |
| **Total Tau** |  |  |  |  |  |  |  | 1 | -0.004  (0.981) | -0.02 (0.874) | 0.09  (0.595) | -0.03  (0.851) | 0.03  (0.825) | -0.12  (0.409) | 0.07  (0.645) | 0.05  (0.712) | 0.06  (0.656) | -0.004  (0.976) |
| **NFL** |  |  |  |  |  |  |  |  | 1 | 0.39  (0.005)** | -0.25  (0.162) | -0.13  (0.459) | -0.14  (0.356) | -0.23  (0.167) | 0.10  (0.502) | -0.05  (0.744) | 0.29  (0.043)* | -0.11  (0.444) |
| **GFAP** |  |  |  |  |  |  |  |  |  | 1 | -0.22  (0.155) | -0.12  (0.417) | -0.11  (0.421) | -0.04  (0.808) | -0.05  (0.714) | 0.03  (0.801) | -0.06  (0.659) | 0.07  (0.592) |
| **IL-2** |  |  |  |  |  |  |  |  |  |  | 1 | 0.53  (<0.001)*** | 0.73  (<0.001)*** | 0.22  (0.184) | 0.41  (0.006)** | -0.10  (0.550) | -0.06  (0.732) | 0.48  (0.001)** |
| **TNF-α** |  |  |  |  |  |  |  |  |  |  |  | 1 | 0.25 (0.094) | 0.34  (0.030)* | 0.13  (0.407) | -0.08  (0.582) | -0.09  (0.549) | 0.30 (0.035)* |
| **IL-1α** |  |  |  |  |  |  |  |  |  |  |  |  | 1 | 0.23  (0.095) | 0.55  (<0.001)*** | 0.12  (0.387) | 0.26  (0.056) | 0.33  (0.010)* |
| **IL-8** |  |  |  |  |  |  |  |  |  |  |  |  |  | 1 | 0.08  (0.584) | -0.09  (0.528) | -0.06  (0.659) | 0.20  (0.162) |
| **IL-12p70** |  |  |  |  |  |  |  |  |  |  |  |  |  |  | 1 | 0.16  (0.255) | 0.35  (0.008)** | 0.009  (0.950) |
| **MCP-1** |  |  |  |  |  |  |  |  |  |  |  |  |  |  |  | 1 | 0.08  (0.555) | -0.22  (0.088) |
| **VEGF-A** |  |  |  |  |  |  |  |  |  |  |  |  |  |  |  |  | 1 | -.003  (0.842) |
| **βNGF** |  |  |  |  |  |  |  |  |  |  |  |  |  |  |  |  |  | 1 |

SUPPLEMENTARY TABLE 2: Spearman correlation matrix and p-values of blood-based biomarkers and fatigue in childhood cancer survivors

|  | **BDNF** | **Total Tau** | **NfL** | **GFAP** | **βNGF** | **IL-2** | **TNF-α** | **IL-1α** | **IL-8** | **IL-12p70** | **MCP-1** | **VEGF** | **General Fatigue** | **Sleep / rest Fatigue** | **Cognitive Fatigue** | | **Total Fatigue** |
| --- | --- | --- | --- | --- | --- | --- | --- | --- | --- | --- | --- | --- | --- | --- | --- | --- | --- |
| **BDNF** | 1 | 0.18 (0.182) | 0.03 (.974) | -0.08 (0.519) | 0.16 (0.237) | 0.07 (0.674) | 0.04 (0.808) | 0.20 (0.140) | -0.13 (0.376) | -0.02 (0.905) | 0.38 (0.003)** | 0.30 (0.021)* | 0.10 (0.434) | -0.14 (0.281) | -0.13 (0.313) | -0.06 (0.613) | |
| **Total Tau** |  | 1 | 0.09 (.981) | -0.02 (0.874) | -0.01 (.976) | 0.08 (0.595) | -0.03 (0.851) | 0.03 (0.825) | -0.12 (0.409) | 0.07 (0.645) | 0.05 (0.712) | 0.06 (0.656) | -0.06 (0.641) | 0.11 (0.377) | 0.01 (0.908) | 0.04 (0.752) | |
| **NfL** |  |  | 1 | 0.39 (0.005)** | -0.11 (.444) | -0.25 (0.162) | -0.13 (0.459) | -0.14 (0.356) | -0.23 (0.167) | 0.10 (0.502) | -0.05 (0.744) | 0.29 (0.043)* | -0.01 (0.974) | 0.07 (0.590) | 0.04 (0.796) | 0.07 (0.602) | |
| **GFAP** |  |  |  | 1 | 0.07 (.592) | -0.22 (0.155) | -0.12 (0.417) | -0.11 (0.421) | -0.03 (0.808) | -0.05 (0.714) | 0.03 (0.801) | -0.06 (0.659) | 0.18 (0.135) | 0.18 (0.128) | 0.11 (0.349) | 0.19 (0.101) | |
| **βNGF** |  |  |  |  | 1 | 0.48 (0.001)** | 0.30 (0.035)* | 0.33 (0.010)* | 0.20 (0.162) | 0.01 (0.950) | -0.22 (0.088) | -0.03 (0.842) | 0.06 (0.626) | -0.02 (0.856) | 0.04 (0.758) | 0.01 (0.953) | |
| **IL-2** |  |  |  |  |  | 1 | 0.53 (<0.001)*** | 0.73 (<0.001)*** | 0.22 (0.184) | 0.41 (0.006)** | -0.09 (0.550) | -0.06 (0.732) | 0.11 (0.443) | 0.17 (0.238) | 0.06 (0.664) | 0.14 (0.328) | |
| **TNF-α** |  |  |  |  |  |  | 1 | 0.25 (0.094) | 0.33 (0.030)* | 0.13 (0.407) | -0.08 (0.582) | -0.09 (0.549) | -0.03 (0.848) | -0.05 (0.734) | -0.06 (0.651) | -0.04 (0.784) | |
| **IL-1α** |  |  |  |  |  |  |  | 1 | 0.23 (0.095) | 0.55 (<0.001)*** | 0.12 (0.387) | 0.25 (0.056) | 0.06 (0.649) | -0.10 (0.438) | 0.01 (0.932) | 0.01 (0.992) | |
| **IL-8** |  |  |  |  |  |  |  |  | 1 | 0.08 (0.584) | -0.09 (0.528) | -0.06 (0.659) | 0.20 (0.143) | -0.09 (0.486) | 0.16 (0.225) | 0.14 (0.288) | |
| **IL-12p70** |  |  |  |  |  |  |  |  |  | 1 | 0.15 (0.255) | 0.35 (0.008)** | -0.05 (0.695) | 0.10 (0.438) | -0.11 (0.419) | -0.05 (0.726) | |
| **MCP-1** |  |  |  |  |  |  |  |  |  |  | 1 | 0.08 (0.555) | 0.13 (0.301) | 0.08 (0.494) | 0.10 (0.429) | 0.13 (0.300) | |
| **VEGF** |  |  |  |  |  |  |  |  |  |  |  | 1 | 0.10 (0.447) | 0.10 (0.443) | -0.19 (0.123) | -0.04 (0.731) | |
| **General Fatigue** |  |  |  |  |  |  |  |  |  |  |  |  | 1 | 0.57 (<0.001)*** | 0.48 (<0.001)*** | 0.80 (<0.001)*** | |
| **Sleep / rest Fatigue** |  |  |  |  |  |  |  |  |  |  |  |  |  | 1 | 0.35 (0.001)*** | 0.75 (<0.001)*** | |
| **Cognitive Fatigue** |  |  |  |  |  |  |  |  |  |  |  |  |  |  | 1 | 0.82 (<0.001)*** | |
| **Total Fatigue** |  |  |  |  |  |  |  |  |  |  |  |  |  |  |  | 1 | |

SUPPLEMENTARY TABLE 3: Spearman correlation matrix and p-values of blood-based biomarkers and fatigue domains in healthy controls

|  | **BDNF** | **Total Tau** | **NfL** | **GFAP** | **βNGF** | **IL-2** | **TNF-α** | **IL-1α** | **IL-8** | **IL-12p70** | **MCP-1** | **VEGF** | **General Fatigue** | **Sleep / rest Fatigue** | **Cognitive Fatigue** | **Total Fatigue** |
| --- | --- | --- | --- | --- | --- | --- | --- | --- | --- | --- | --- | --- | --- | --- | --- | --- |
| **BDNF** | 1 | -0.25 (0.304) | 0.01 (0.996) | -0.13 (0.556) | 0.14 (0.507) | -0.16 (0.471) | -0.16 (0.463) | -0.20 (0.316) | 0.23 (0.348) | -0.26 (0.263) | 0.31 (0.164) | 0.58 (0.002)** | -0.07 (0.721) | 0.11 (0.577) | 0.580 (0.025)* | -0.14 (0.492) |
| **Total Tau** |  | 1 | 0.51 (0.020)* | 0.22 (0.355) | -0.21 (0.369) | -0.34 (0.184) | 0.13 (0.606) | 0.03 (0.910) | -0.54 (0.031)* | 0.16 (0.505) | 0.24 (0.336) | 0.04 (0.854) | -0.11 (0.652) | -0.33 (0.157) | 0.25 (0.281) | -0.07 (0.757) |
| **NfL** |  |  | 1 | 0.51 (0.005)** | -0.31 (0.093) | -0.39 (0.044)* | -0.32 (0.104) | -0.08 (0.661) | -0.42 (0.052) | 0.16 (0.422) | 0.25 (0.248) | 0.08 (0.651) | -0.15 (0.451) | -0.04 (0.858) | -0.16 (0.419) | -0.13 (0.519) |
| **GFAP** |  |  |  | 1 | -0.22 (0.255) | -0.47 (0.020)* | -0.10 (0.619) | 0.10 (0.637) | 0.03 (0.887) | 0.09 (0.680) | -0.30 (0.182) | 0.24 (0.216) | -0.22 (0.300) | -0.22 (0.299) | -0.14 (0.517) | -0.24 (0.244) |
| **βNGF** |  |  |  |  | 1 | 0.26 (0.196) | 0.28 (0.143) | 0.27 (0.145) | 0.24 (0.282) | 0.08 (0.700) | -0.03 (0.907) | 0.08 (0.670) | -0.03 (0.885) | 0.15 (0.475) | -0.22 (0.284) | -0.05 (0.810) |
| **IL-2** |  |  |  |  |  | 1 | 0.77 (<0.001)*** | 0.74 (<0.001)*** | 0.15 (0.529) | 0.31 (0.148) | -0.29 (0.205) | -0.24 (0.238) | 0.04 (0.855) | 0.12 (0.605) | 0.27 (0.227) | 0.11 (0.620) |
| **TNF-α** |  |  |  |  |  |  | 1 | 0.57 (0.002)** | 0.51 (0.019)* | 0.30 (0.151) | -0.40 (0.056) | 0.16 (0.433) | 0.27 (0.203) | 0.01 (0.971) | 0.31 (0.143) | 0.23 (0.282) |
| **IL-1α** |  |  |  |  |  |  |  | 1 | 0.15 (0.503) | 0.76 (<0.001)*** | -0.07 (0.764) | 0.19 (0.323) | 0.11 (0.592) | 0.13 (0.524) | 0.29 (0.148) | 0.16 (0.426) |
| **IL-8** |  |  |  |  |  |  |  |  | 1 | -0.02 (0.943) | -0.42 (0.075) | 0.57 (0.006)** | 0.08 (0.740) | 0.08 (0.736) | -0.21 (0.391) | -0.03 (0.892) |
| **IL-12p70** |  |  |  |  |  |  |  |  |  | 1 | 0.13 (0.579) | -0.01 (0.999) | 0.22 (0.317) | 0.09 (0.695) | 0.23 (0.307) | 0.18 0(.431) |
| **MCP-1** |  |  |  |  |  |  |  |  |  |  | 1 | 0.29 (0.173) | 0.05 (0.842) | 0.14 (0.541) | 0.03 (0.892) | 0.06 (0.812) |
| **VEGF** |  |  |  |  |  |  |  |  |  |  |  | 1 | -0.12 (0.546) | -0.07 (0.735) | -0.24 (.0209)* | -0.15 (0.447) |
| **General Fatigue** |  |  |  |  |  |  |  |  |  |  |  |  | 1 | 0.60 (0.001)** | 0.47 (0.011)* | 0.92 (<0.001)*** |
| **Sleep / rest Fatigue** |  |  |  |  |  |  |  |  |  |  |  |  |  | 1 | 0.23 (0.245) | 0.77 (<0.001)*** |
| **Cognitive Fatigue** |  |  |  |  |  |  |  |  |  |  |  |  |  |  | 1 | 0.60 (0.001)** |
| **Total Fatigue** |  |  |  |  |  |  |  |  |  |  |  |  |  |  |  | 1 |

SUPPLEMENTARY FIGURE 2: Biomarker concentrations in fatigued vs. non-fatigued childhood cancer survivors across four fatigue domains.


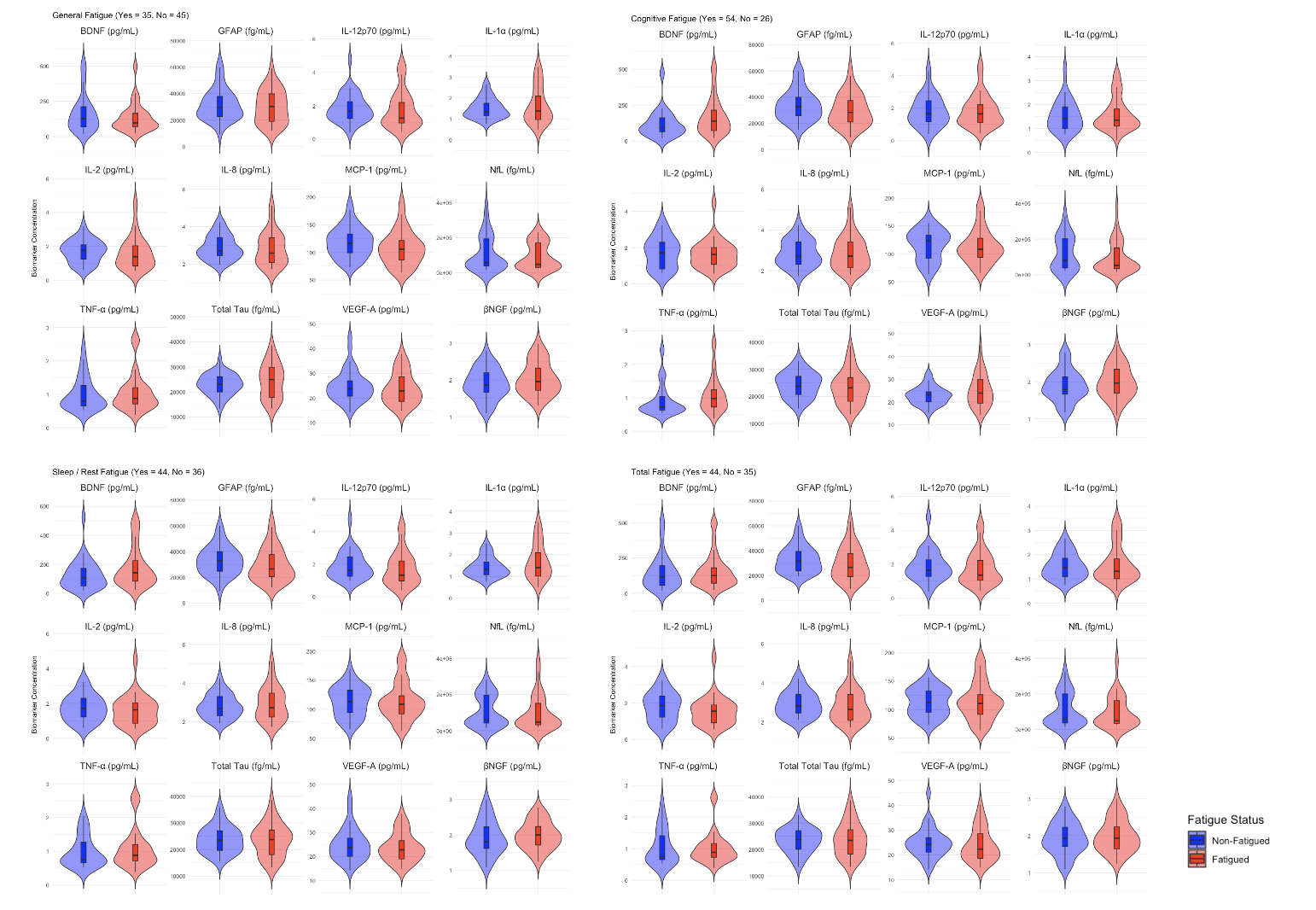


Note. Violin plots display the distribution of 12 blood-based biomarkers (BDNF, βNGF, GFAP, IL-12p70, IL-1α, IL-2, IL-8, MCP-1, NfL, TNF-α, Total Tau, VEGF) comparing fatigued and non-fatigued CCS for each fatigue domain as measured by the PedsQL-MFS: General Fatigue, Sleep/Rest Fatigue, Cognitive Fatigue, and Total Fatigue. Fatigue status was determined by z-scores (< -1 indicating “fatigued”). Blue indicates non-fatigued participants, red indicates fatigued participants.

SUPPLEMENTARY FIGURE 3: Silhouette analysis to determine the optimal number of clusters based on biomarker profiles in childhood cancer survivors

Note. The silhouette method was applied to evaluate clustering quality for 1 to 10 clusters using standardized biomarker data. The silhouette score quantifies how well each participant fits within its assigned cluster, with higher scores indicating better-defined clusters. A clear peak at *k* = 2 suggests that a two-cluster solution best fits the data, supporting the presence of two distinct biomarker-based CCS subgroups.


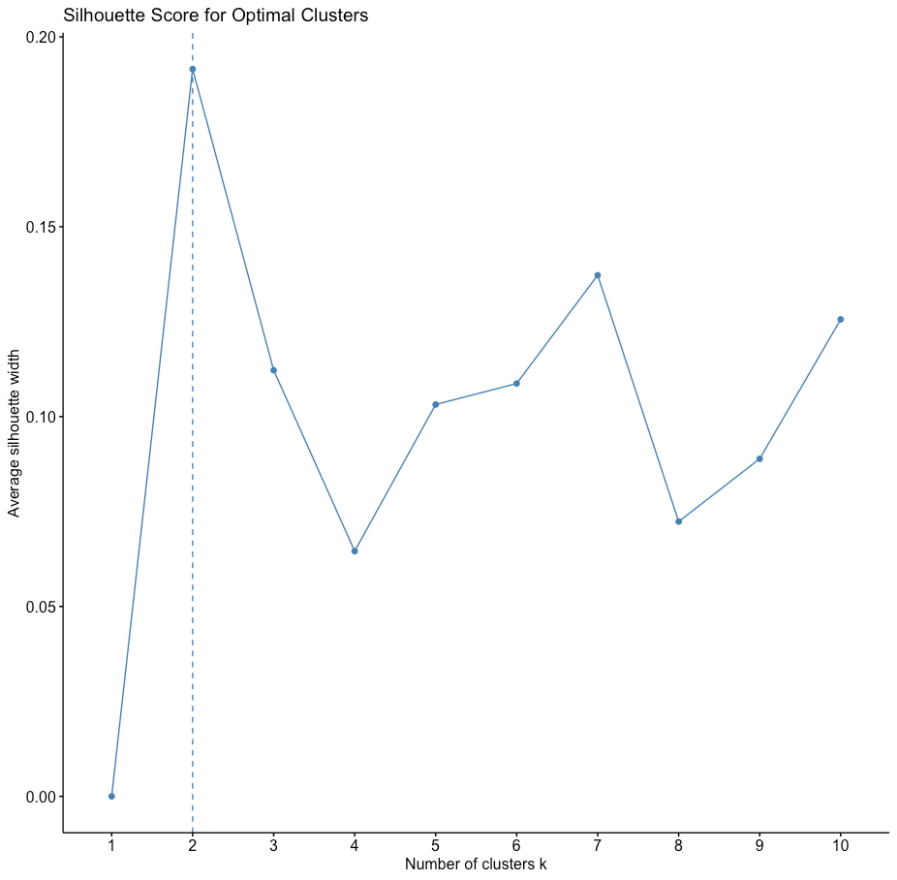


SUPPLEMENTARY TABLE 4: Spearman correlation coefficients ρ (and p-values) for associations between blood-based biomarkers and fatigue domains within each biomarker-defined cluster (Cluster 1 and Cluster 2) of childhood cancer survivors

| **CLUSTER 1** | | | | |
| --- | --- | --- | --- | --- |
|  | **General Fatigue** | **Sleep / Rest Fatigue** | **Cognitive Fatigue** | **Total Fatigue** |
| **BDNF** | 0.01 (0.94) | -0.19 (0.21) | -0.07 (0.67) | -0.09 (0.56) |
| **GFAP** | 0.13 (0.38) | -0.27 (0.04)* | 0.20 (0.16) | -0.26 (0.03)* |
| **IL-12p70** | -0.05 (0.75) | 0.18 (0.26) | -0.20 (0.21) | -0.12 (0.47) |
| **IL-1α** | 0.15 (0.34) | 0.03 (0.86) | -0.12 (0.44) | 0.01 (0.99) |
| **IL-2** | 0.05 (0.79) | 0.11 (0.54) | -0.06 (0.75) | 0.01 (0.95) |
| **IL-8** | 0.14 (0.42) | -0.27 (0.10) | -0.01 (0.99) | 0.02 (0.93) |
| **MCP-1** | 0.18 (0.25) | 0.14 (0.38) | 0.17 (0.27) | 0.18 (0.24) |
| **NfL** | -0.02 (0.89) | 0.17 (0.34) | 0.18 (0.32) | 0.17 (0.36) |
| **Total Tau** | -0.01 (0.93) | 0.06 (0.68) | 0.13 (0.40) | 0.06 (0.68) |
| **TNF-α** | -0.20 (0.23) | -0.14 (0.41) | -0.28 (0.05) | -0.26 (0.11) |
| **VEGF** | 0.15 (0.31) | 0.12 (0.42) | -0.13 (0.38) | 0.01 (0.98) |
| **βNGF** | 0.04 (0.79) | -0.08 (0.57) | 0.04 (0.81) | 0.01 (0.99) |
| **CLUSTER 2** | | | | |
|  | **General Fatigue** | **Sleep / Rest Fatigue** | **Cognitive Fatigue** | **Total Fatigue** |
| **BDNF** | 0.25 (0.27) | -0.01 (0.99) | -0.13 (0.58) | 0.004 (0.99) |
| **GFAP** | 0.26 (0.23) | -0.02 (0.91) | -0.02 (0.93) | 0.08 (0.72) |
| **IL-12p70** | -0.36 (0.13) | -0.16 (0.51) | -0.24 (0.31) | -0.31 (0.19) |
| **IL-1α** | -0.42 (0.03)* | -0.38 (0.04)* | -0.06 (0.80) | -0.34 (0.13) |
| **IL-2** | -0.09 (0.75) | 0.40 (0.14) | 0.40 (0.14) | 0.29 (0.29) |
| **IL-8** | 0.30 (0.21) | 0.26 (0.27) | -0.48 (0.02)* | -0.41 (0.04)* |
| **MCP-1** | 0.08 (0.73) | 0.03 (0.89) | -0.02 (0.92) | 0.02 (0.93) |
| **NfL** | 0.05 (0.82) | -0.03 (0.88) | -0.11 (0.64) | -0.08 (0.74) |
| **Total Tau** | -0.16 (0.49) | 0.20 (0.39) | -0.22 (0.33) | -0.04 (0.85) |
| **TNF-α** | -0.40 (0.14) | -0.40 (0.14) | -0.49 (0.04)* | -0.49 (0.04)* |
| **VEGF** | -0.05 (0.83) | 0.10 (0.68) | -0.31(0.18) | -0.15 (0.51) |
| **βNGF** | 0.07 (0.76) | 0.12 (0.60) | 0.09 (0.69) | 0.03 (0.89) |

SUPPLEMENTARY FIGURE 4: Group comparisons of fatigue and biomarker levels in childhood cancer survivor (CCS) clusters and healthy controls (HCs).


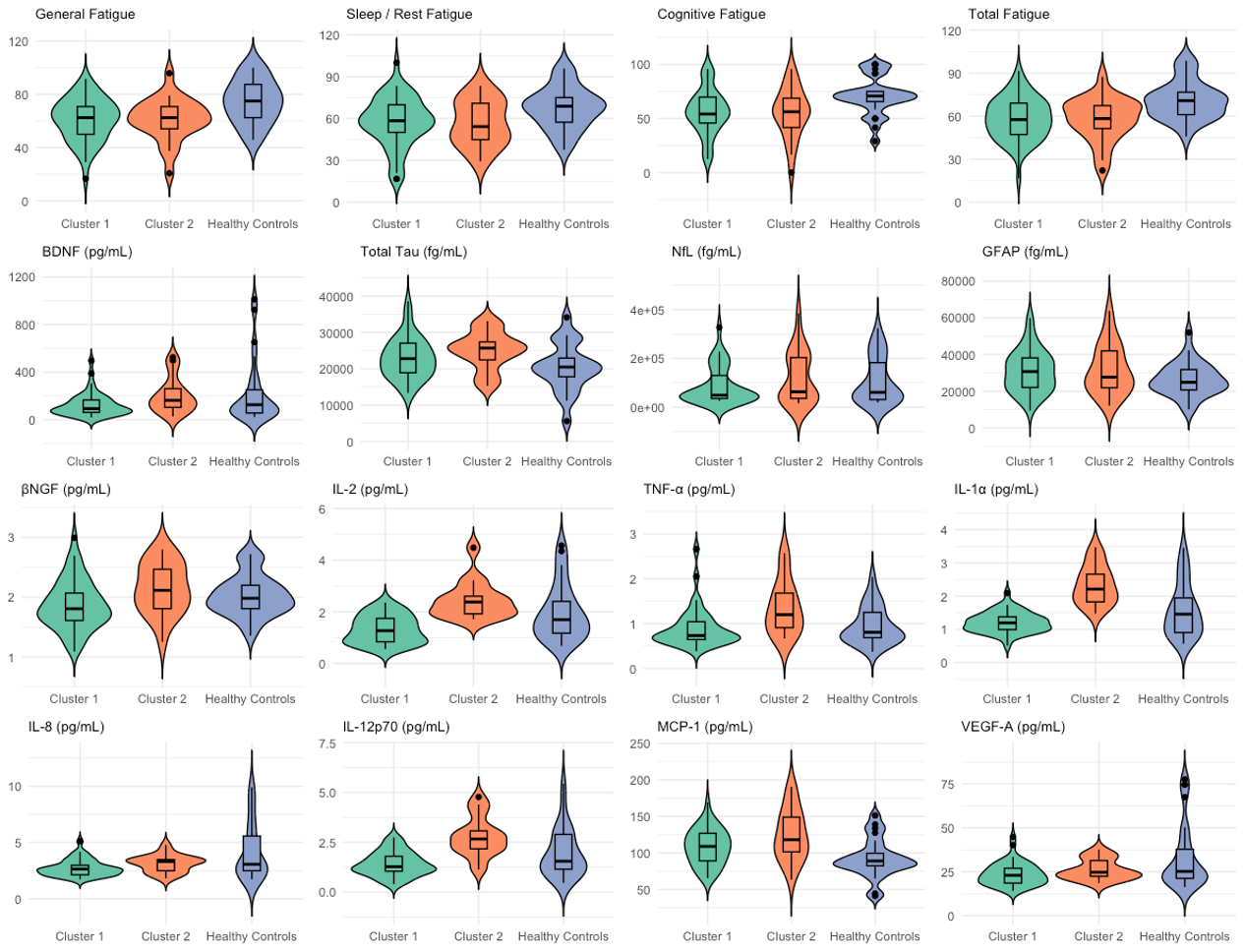


Violin plots with embedded boxplots showing the distribution of fatigue scores (General, Sleep/Rest, Cognitive, Total) and biomarker concentrations across Cluster 1, Cluster 2, and Healthy Controls. Clusters were defined using k-means clustering on standardized biomarker data in childhood cancer survivors. Fatigue scores are derived from the PedsQL-MFS, ranging from 0 to 100, with higher scores indicating lower fatigue. Biomarker concentrations are displayed in their respective units. Density curves are trimmed to the observed data range. Group colors represent Cluster 1 (green), Cluster 2 (orange), and Healthy Controls (purple).
